# Drive-By Bridge Frequency Identification under Operational Roadway Speeds Employing Frequency Independent Underdamped Pinning Stochastic Resonance (FI-UPSR)

**DOI:** 10.3390/s18124207

**Published:** 2018-11-30

**Authors:** Ahmed Elhattab, Nasim Uddin, Eugene OBrien

**Affiliations:** 1 Department of Civil, Construction, and Environmental Engineering, The University of Alabama at Birmingham, 1075 13th St S, Birmingham, AL 35205, USA; nuddin@uab.edu; 2 School of Civil Engineering, University College Dublin, Newstead Block B, Belfield, Dublin D04V1W8, Ireland; eugene.obrien@ucd.ie

**Keywords:** stochastic resonance, drive-by bridge inspection, structural health monitoring SHM, BSHM, damage detection, frequency independent stochastic resonance

## Abstract

Recently, drive-by bridge inspection has attracted increasing attention in the bridge monitoring field. A number of studies have given confidence in the feasibility of the approach to detect, quantify, and localize damages. However, the speed of the inspection truck represents a major obstacle to the success of this method. High speeds are essential to induce a significant amount of kinetic energy to stimulate the bridge modes of vibration. On the other hand, low speeds are necessary to collect more data and to attenuate the vibration of the vehicle due to the roughness of the road and, hence, magnify the bridge influence on the vehicle responses. This article introduces Frequency Independent Underdamped Pinning Stochastic Resonance (FI-UPSR) as a new technique, which possesses the ability to extract bridge dynamic properties from the responses of a vehicle that passes over the bridge at high speed. Stochastic Resonance (SR) is a phenomenon where feeble information such as weak signals can be amplified through the assistance of background noise. In this study, bridge vibrations that are present in the vehicle responses when it passes over the bridge are the feeble information while the noise counts for the effect of the road roughness on the vehicle vibration. UPSR is one of the SR models that has been chosen in this study for its suitability to extract the bridge vibration. The main contributions of this article are: (1) introducing a Frequency Independent-Stochastic Resonance model known as the FI-UPSR and (2) implementing this model to extract the bridge vibration from the responses of a fast passing vehicle.

## 1. Introduction

The degradation in the structural integrity of highway bridges is attributed to aging and significant increases in freight volumes. In the United States, approximately 11% of bridges on the transport network has been classified as structurally defective [1]. This percentage is expected to spike at 25% within a ten-year period [1]. Notably, the most recent federal estimate for the cost of bridge rehabilitation projects is found to be $123 billion [2]. Therefore, the structural safety assessment of bridges on the road network has become an essential area of research. To this end, the detection of incipient defects has become a crucial issue where early detection and repair of damage could allow for prioritization of resources, reduce the retrofitting budget, and, simultaneously, preserve the functionality of the network. This has led the field of Structural Health Monitoring (SHM) identifying the condition of existing structures using sensor readings [3,4,5,6,7,8,9,10]. Conventional Structural Health Monitoring techniques require onsite instrumentation, which has disadvantages (e.g., necessity for a continuous source of power, data acquisition, and transmission electronics as well as sensor installation and maintenance) that have slowed their uptakes [11,12]. This is in addition to the impact on the capacity of the roadway due to the associated closure of bridge lanes [13,14,15]. Recently, researchers have investigated the feasibility of moving the instrumentation from the bridge structure to a passing vehicle to assess the bridge health condition, which has been referred to as “Drive-by Bridge Inspection” [16]. Yang et al. [17,18] were the first to publish a preliminary study investigating the feasibility of the approach. The researchers developed a mathematical expression for the Vehicle-Bridge Interaction (VBI) problem to explore the extraction of the fundamental bridge frequency from the vehicle’s response. They found that the response of the vehicle includes four main frequencies: (1) the driving frequency *v*/*L* (Hz) (where ‘*v*’ is the speed of the vehicle and ‘*L*’ is the bridge span), (2) the vehicle frequency *f_v_* (Hz) and (3) two frequencies, which are shifted by a value equal to half of the driving frequency from the bridge first natural frequency, *f_b_*, i.e., $f_{b}\pm \frac{v}{2L}$ (Hz). Furthermore, they declared that higher speeds have a positive impact on the approach since they increase the amplitude of the bridge frequency in the spectrum and, hence, increase the visibility of the bridge frequency components. In the same vein, they investigated utilizing the vehicle responses in detecting the change in the bridge damping ratio. They observed a significant drop in the spectrum power as the bridge damping ratio increased. The findings of this study suggested that the approach is feasible.

Afterwards, a number of studies were carried out studying the variation in the acceleration spectra of the vehicle response as a measure of bridge deterioration in the presence of the road roughness [16,19,20,21,22,23,24]. These found that road roughness induces a considerable vibration in the vehicle suspension system, which results in the signal being overwhelmed by the dynamic characteristics of the vehicle. These works concluded that the use of lower speeds for the inspection vehicle provides more data, attenuates the road roughness effect on the recorded signal, and, hence, the bridge responses become evident in the signal. In a study shown in Reference [6], the effect of road roughness on the vehicle response was substantially removed theoretically using a half car model by subtracting the acceleration signals of two consecutive axles. Under high speed, the bridge frequency has dominated the response spectrum of the subtracted signal. Elhattab et al. [25,26] have investigated the concept using an explicit VBI solver, which is included in a commercial Finite Element Program (LS-Dyna) and similar findings were observed. The approach was confined to numerical simulations and no field demonstration has been carried out. To probe the fidelity of the drive-by approach, Lin and Yang [27] utilized field test data to obtain the natural frequency of the Da-Wu-Lun Bridge in Taiwan from passing vehicle responses. They reported a successful identification of the bridge frequency for speeds below 40 km/h (24.85 mph). For higher speeds, the bridge frequency was swamped by vibrations arising from the roughness of the road. Oshima, Yamaguchi [28] showed the effectiveness of a vehicle equipped with a vibrator in extracting bridge frequencies. However, they recommended using lower speeds to attain higher resolution. 

In summary, previous studies have identified the speed of the inspection vehicle as a key challenge to accurately identify the bridge frequencies. When the speed is increased, the vehicle’s excitation is increased due to the road roughness. Thus, the bridge response is less prominent due to excessive vehicle vibration. This article is devoted to the magnification of the feeble response of the bridge in the vehicle signal utilizing the Stochastic Resonance (SR) phenomenon. 

Stochastic Resonance (SR) is a process of weak response detection in a signal overwhelmed by a global system response. It is one of the established mathematical models for nonlinear systems where generally feeble input information (such as a weak signal) can be amplified and optimized by exploiting the noise in the signal. The approach was proposed in 1981 by Benzi, Sutera [29] as an explanation of the observed periodicity in the ice ages on earth. Since then, the approach has spurred interest and many researchers have investigated the application of this hypothesis in different disciplines [30]. SR was originally built to work for bi-stable/overdamped systems. Bi-stable systems are systems in which the feeble information is oscillating between two stable positions. The overdamped term implies that the observed system’s stiffness is negligible in comparison with the surrounding domain. To illustrate the essence of these two underlying terminologies, a common example of SR implementation by crickets will be discussed. A cricket’s hair cells receive a signal from the surrounding noisy environment. Then they apply the SR technique to explore the possibility of a bird attack [31]. In this example, the feeble information is the periodic air pressure change caused by the predator’s wings, which varies between two stable positions (positive and negative as the wings go up and down). Since the influence of the pressure perturbation around the bird’s wing on the surrounding domain is negligible, the system is an overdamped one. Several studies have investigated the cooperative effect of noise in bi-stable/overdamped systems in detecting weak signals corrupted by a heavy background noise [32,33,34,35,36,37,38,39]. Following the publication of these work, researchers have investigated employing SR for tri-stable [40,41,42] and multi-stable [43] systems and underdamped/bi-stable systems [44,45,46,47,48]. These methods require a priori definition of the targeted frequency to extract the weak signal. Wang, He [49] introduced the Adaptive Multiscale Noise Tuning SR (AMSTSR) technique, which does not require prior knowledge of the targeted frequency. However, the approach is built for bi-stable systems. Recently, Zhang, He [50] introduced an SR model with the capability to detect mono-stable (i.e., the weak property is stable at one particular point) and bi-stable systems known as Underdamped Pinning Stochastic Resonance (UPSR). However, the approach requires identifying the frequency of interest in advance.

This paper is intended to pave the way for the exploitation of SR in the Civil Structural Health Monitoring domain with an emphasis on Drive-by Bridge Inspection. Presuming that most Civil Engineering structures are lightly dampened in nature and are mostly mono-stable systems (i.e., the structure tends to stabilize at a certain mode of vibration), the authors herein adopt the UPSR model for its suitability to the problem under study. The article will look at potential synergies of incorporating visual demarcation with the UPSR technique to obviate the need to define the frequency of weak signals. The proposed visualization approach will facilitate the observation of the SNR attribute of signals that lie in a specified frequency band where SNR (i.e., high SNR value) is exploited as a metric to discriminate between feeble signals and the background noise. The model stemming from this step will be introduced as Frequency Independent-UPSR (FI-UPSR). The proposed technique will be used to detect weak signals in a record disrupted by heavy noise. Afterwards, the approach will be implemented to extract bridge frequency from the response of a fast vehicle. The approach will be explored numerically using a VBI MATLAB model and experimentally using a full-scale field test.

The paper is organized as follows: Section 2 introduces Frequency Independent-UPSR (FI-UPSR) and the proposed visual demarcation approach. Section 3 demonstrates numerically the implementation of FI-UPSR in extracting bridge frequencies from the responses of a quickly passing vehicle. Lastly, a full-scale field test data is used to examine the fidelity of the proposed approach in the field. This will be presented in Section 4.

## 2. Frequency Independent Underdamped Pinning Stochastic Resonance (FI-UPSR)

### 2.1. Background to Underdamped Pinning Stochastic Resonance (UPSR)

**Figure 1:**
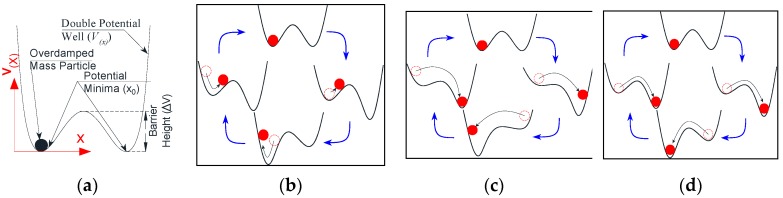
Conventional Overdamped Bi-stable Stochastic Resonance Model. (**a**) Model terminology and (**b**) particle perturbation by the force *f* (**c**) particle perturbation by the force *f* and noise *D* in the general case. (**d**) Particle perturbation by the force *f* and the noise *D* when resonance is approached.

The principle underlying the Stochastic Resonance phenomenon can be explained as follows: consider a heavily damped mass moving in a symmetric double potential well, *V*(*x*), as shown in Figure 1a. The mass is moving due to a fluctuating force *f*(*t*) contaminated by an additive noise having intensity, *D*. The application of a weak periodic force *f*(*t*) will tilt the potential well up and down. However, with a low intensity of force, it is not big enough to let the particle surmount the potential barrier and move from one potential minima to the other (Figure 1b). In contrast, the noise induces a more intense hopping for the particle around the potential minima (Figure 1c). By tuning the noise periodicity (or changing the potential geometry, *V*(*x*)), the hopping induced by the noise can be synchronized with the weak periodic force (Figure 1d) [30]. Since noise in most cases is the uncontrollable term, the SR adjusts the shape of its potential well (*V*(*x*)) to synchronize the hopping between potential minima by changing the parameters of the potential well. Within this framework, the weak periodic force *f*(*t*) refers to the targeted feeble information, which is the input weak signal (*s*(*t*)). This signal is swamped by a severe background noise (*n*(*t*)). The extraction of this feeble signal will be done by resonating the hopping between the two potential minima through changes in the parameters of the potential well (*V*(*x*)). The position of the particle at resonance (*x*(*t*)) represents the extracted feeble feature (herein the extracted weak signal), which is amplified when the hopping between potential minima is synchronized. The dynamic equation of this type of SR is presented in Equation (1).

(1) $$\frac{d^{2}x}{dt^{2}}=-V^{\prime }\left(x\right)-\gamma \frac{dx}{dt}+\left\{s\left(t\right)+n\left(t\right)\right\}$$

where x(t) is the extracted weak signal, ${V^{\prime }}_{\left(x\right)}$ is the first derivative of the potential well (*V(x)*), γ is the system damping, and {s(t)+n(t)} is the input signal added with noise. Equation (1) implies that the Stochastic Resonance phenomenon is a single degree of freedom filtering process where feeble signals are amplified (x(t)) by approaching resonance. There exists a plethora of models to describe different types of potential shapes. Herein, we adopt the UPSR model for its suitability to the problem understudy.

The potential function of the UPSR model [50] is shown below.

(2) $$V\left(x\right)=V_{0}-V_{d}\left(\exp\left(-\frac{{\left(x+x0\right)}^{2}}{L^{2}}\right)+\exp\left(-\frac{{\left(x-x0\right)}^{2}}{L^{2}}\right)\right)$$

where $V_{0}$ is a constant and it will not be considered since it will have no effect after the function is differentiated, as shown in Equation (1), $V_{d}$ is the depth of the pinning, *L* is the length between the two pinnings, and $\pm x_{0}$ is the center of each pinning. The potential function of the UPSR is governed by three parameters (i.e., $V_{d}$, *L*, and $x_{0}$) that provide effective representation for mono-stable and bi-stable systems. Zhang, He [50] provide a numerical discretization for Equation (1) considering the potential function given in Equation (2), which is presented in Appendix A. As previously noted, to extract the weak signal (x(t)), the potential function is tuned until the noise is synchronized with the weak signal. In other words, when Equation (2) approaches resonance. At this point (i.e., when the signal and the noise are tuned), the extracted signal will have the highest Signal to Noise Ratio (SNR). Therefore, the discretized version of Equation (1) (i.e., Equation (A1) presented in Appendix A) will be solved several times for different potential parameters (i.e., *V_d_*, *L*, and $x_{0}$) until this condition is achieved. The selection for the parameter ranges is done arbitrarily. Therefore, the ranges are changed randomly until the signal of the highest SNR is extracted. This framework is followed due to the lack of knowledge about the mathematical relationship between the values of the selected parameters (i.e., *V_d_*, *L*, and $x_{0}$) and the characteristics of the extracted signal (e.g., frequency and amplitude). This is considered to be one of the major drawbacks of the SR filtering process. Section 2.2 of this article addresses this issue by deriving a direct relationship between the potential parameters of the UPSR model and the frequency of the extracted signal. This relationship is then used to establish a criterion for parameter initialization (i.e., *V_d_*, *L*, and $x_{0}$). 

**Figure 2:**
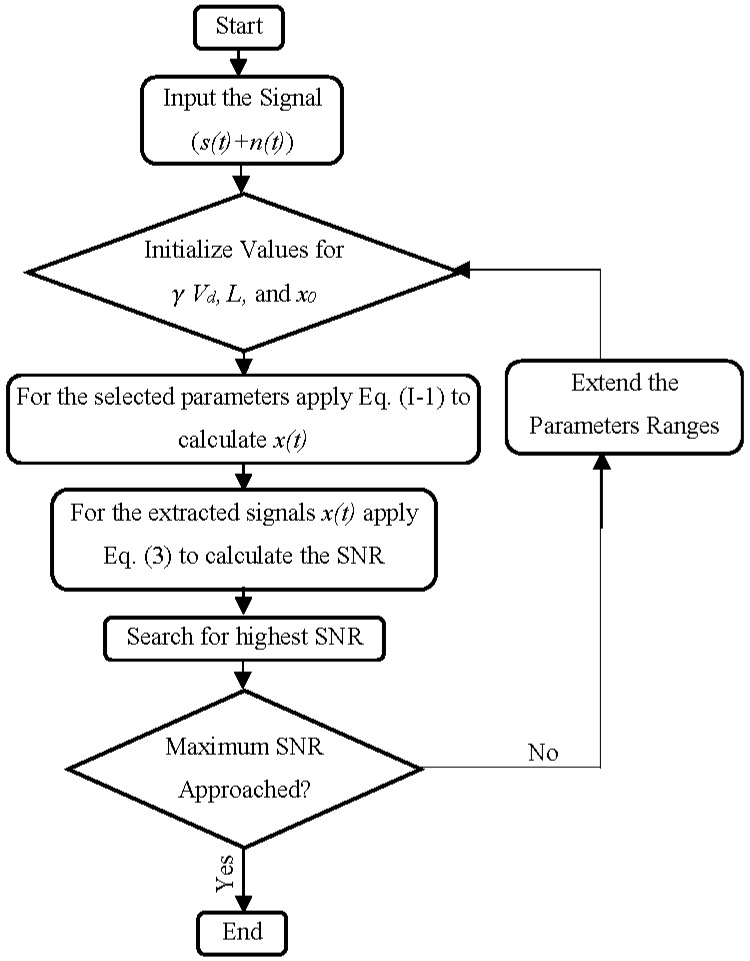
UPSR process for extracting the feeble signal at a specific frequency [50].

The SNR of the extracted signal is calculated below [50].

(3) $$SNR=10\log_{10}\frac{A_{s}}{\sum_{0}^{N/2}A_{i}}$$

where $A_{s}$ is the power of the targeted signal (i.e., the original one, *s*(*t*), rather than the extracted one, *x*(*t*)), and $\sum_{0}^{N/2}A_{i}$ is the summation of the power of the computed signal *x(t).* The UPSR process is summarized in Figure 2. More details of the UPSR method are provided in Reference [50].

The preceding discussion deals with the equations, procedures, and terminologies of the UPSR technique. The following section presents the changes made to make the UPSR approach frequency independent.

### 2.2. Frequency Independent Underdamped Pinning Stochastic Resonance FI-UPSR

The core of the proposed FI-UPSR approach is to move from searching for the optimum potential parameters (i.e., *V_d_*, *L*, and $x_{0}$) that maximize the SNR for a targeted signal to observe the SNR attributable to a specified frequency band where the signal that exhibits a relatively high SNR ratio in this band is the weak signal. To this end, the SNR values are explored in a three-dimensional plot to facilitate the identification of the highest SNR visually. The SNRs are computed by first generating a set of different combinations for the potential parameters. Specifically, at a certain potential depth (*V_d_*), a range of potential shapes are generated (by varying *L* and *x*_0_). For each combination (i.e., *V_d_*, *L*, and $x_{0}$), the corresponding signal is extracted by using Equation (A1) (Appendix A). Afterwards, the SNR of this signal is computed. The calculation of the SNR needs to be independent of the targeted signal power ($A_{s},$ which corresponds to the weak signal (s(t))). Therefore, Equation (3) has been modified to be as follows where $A_{x}$ is the maximum power of the computed signal (x(t)).

(4) $$SNR=10\log_{10}\frac{A_{x}}{\sum_{0}^{N/2}A_{i}}$$

The selection of the potential parameter values and ranges is a substantial contributor to the success of the FI-UPSR approach. As highlighted, the FI-UPSR user will specify the upper and lower limits for the frequency band of interest [*f_min_*→*f_max_*]. Consequently, the corresponding potential parameter values for the selected frequency limits must be computed [${\left\{V_{d},\;L,\;\mathrm{and}\;x_{0}\right\}}_{for\;f_{min}}\to {\left\{V_{d},\;L,\;\mathrm{and}\;x_{0}\right\}}_{for\;f_{max}}$] to generate a set of parameter combinations, which will be utilized to generate the SNR surface plot. However, there is no direct relation between the values of the potential parameters (i.e., *V_d_*, *L*, and *x*_0_) and the frequency of the extracted signal (x(t)), which is one of the main drawbacks of conventional SR filters. Rather than relying on expert judgment on the selection of the proper potential values, we have derived a direct approximate relationship between the potential parameters and the frequency of the extracted signal. 

The relationship is presented in Equation (5) of which the full derivation is provided in Appendix B.

(5) $$\frac{f_{approximate}}{R}=\sqrt{\frac{V_{d}}{\pi^{2}L^{2}}}$$

where *R* is a rescaling factor that is used to rescale the time step in Equation (A1) and *f_approximate_* is the approximate frequency of the extracted signal *x*(*t*). 

The value of the potential depth plays a prominent role in the filtering process. Small potential depth (*V_d_*) provides a small potential barrier. Thus, the particle jumps through the barrier without resistance. As *V_d_* increases, surmounting the potential barrier becomes harder for the particle unless the noise and the force are tuned. As a result, high *V_d_* values block unfavorable noise and provide better extraction for the weak signal. At a certain potential depth *V_d_*, the values of *L* corresponding to a specific frequency range ($f_{1},\;f_{2},\ldots ,f_{n}$) can be computed using Equation (5). Lastly, the values of the pinning centers ($\pm x_{0}$) are related to the values of *L* where: $L\leq \sqrt{2}x_{0}$. This relation will be explained in the following section after visualizing the SNR surface plot. 

The rescaling factor *R* is a function of the time step size or the available sampling frequency and it impacts the accuracy of Equation (A1). The damping term in Equation (1) is used to smoothen the oscillation between the potentials to better recognize resonance when approached. The effect of the values of the rescaling factor *R* and the damping term, γ, on the filtering process will be explored later in the paper. Table 1 provides recommended values for $V_{d}$, *R*, and γ that can be used as an initial trial. The values of *L* are calculated using Equation (5) and the values of $x_{0}$ are calculated using the $L\leq \sqrt{2}x_{0}$ relation.

**Figure 3:**
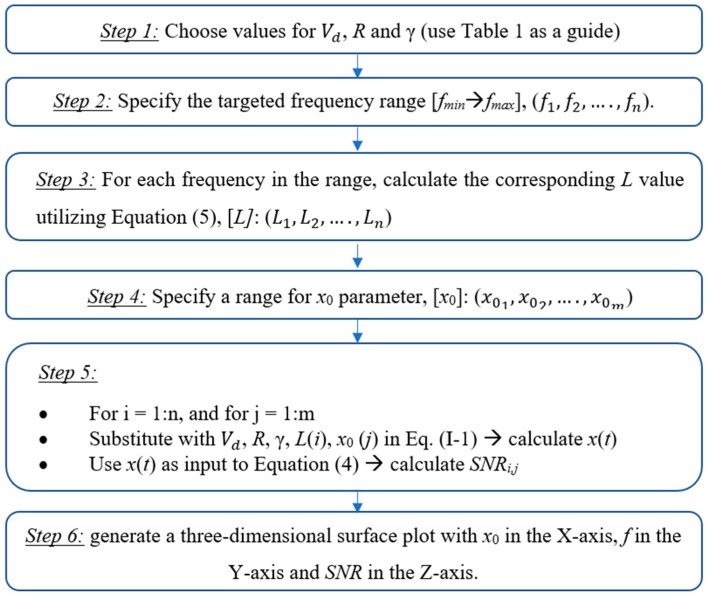
The FI-UPSR procedure.

The FI-UPSR procedure is summarized in the following Figure 3.

### 2.3. Investigating the FI-UPSR Approach

**Figure 4:**
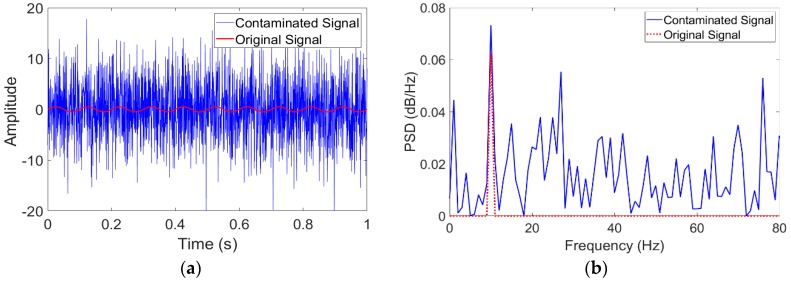
The raw and contaminated signals and their spectra. (**a**) Signals record; (**b**) Signals PSD.

**Figure 5:**
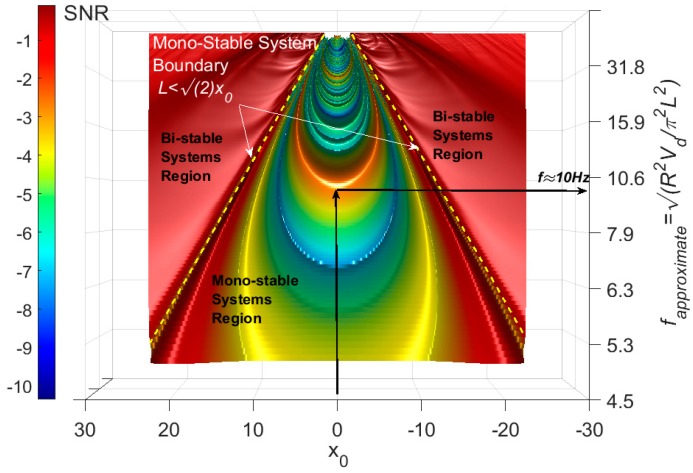
SNR-surface plot for the extracted signals at different potential geometries.

**Figure 6:**
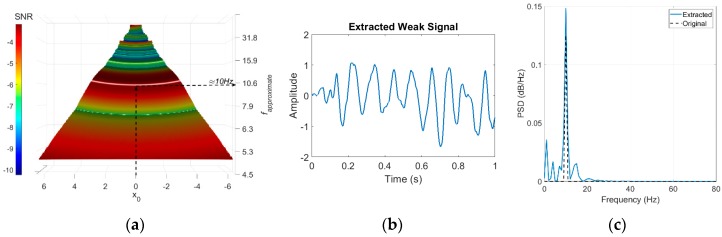
FI-UPSR results (**a**) SNR surface plot, (**b**) extracted weak signal, and (**c**) PSD plot for the extracted signal.

The feasibility of the proposed FI-UPSR method will be examined using a sinusoidal signal (s=Asin(2πft) where *A* = 0.5 and *f* = 10 Hz) contaminated with a Gaussian white noise of −15 dB in power. The signal record and the signal Power Spectral Density (PSD) are shown in Figure 4. It is evident that the signal has been totally disrupted by the noise. The contaminated signal has been processed using the proposed FI-UPSR approach. In this case, the damping coefficient was chosen as γ = 0.5, *V_d_* was 100, the scaling factor, *R* was set to 50, and the sampling frequency, *f_s_* was 2000 Hz. Determining the frequency range is incumbent on the user where it is presumed that the frequency of interest should lie within a range that is known a priori. In this example, the scanned frequency window ranges from 5 to 80 Hz in increments of 0.1 Hz. The limits for the scanned window are chosen so the frequency of the pure signal (*f* = 10 Hz) lies between these limits. The parameter, $x_{0}$, varies from −22.5 to 22.5 in increments of 0.05. The selection of this range will be discussed in detail later in the paper. The SNR surface is plotted by using a perceptual color map that includes hue, illumination, and saturation dimensions to provide a better visualization surface topography, as shown in Figure 5 [51,52]. The map has a luminance dimension, which provides brightness for the crests and darkness for troughs. Visual examination of the figure reveals a number of important characteristics. First, it is evident that the system is symmetric around $x_{0}$ = 0. Another point of note is that the left and the right dark red-colored regions of the figure (the triangles) have SNR values consistently close to zero. This attribute is associated with the geometry of the UPSR potential, which can be reshaped to provide a mono-stable or a bi-stable potential well. Zhang, He [50] showed that a bi-stable model is obtained when $L\leq \sqrt{2}x_{0}$ (boundary depicted in the figure with a dashed yellow line) while a mono-stable model is obtained otherwise. This area is in the first category (i.e., bi-stable model where $L\leq \sqrt{2}x_{0}$). This intriguing feature is essential to implement SR in health monitoring applications since most of the civil structures are mono-stable systems. We will focus on the mono-stable zone rather than visualizing the whole *x*_0_ domain. Figure 6a presents a surface plot of the mono-stable region of Figure 4 after eliminating the bi-stable zones. In the same vein, the SNR plot exhibits a significant peak wave within the boundaries of this mono-stable system where for $x_{0}$ = 0, *f_approximate_* = 10.0 Hz. *f_approximate_* is an approximate frequency for the extracted signal computed using Equation (5), but it does not represent the actual signal frequency. The actual signal frequency is identified by transferring the extracted signal, *x*(*t*), from the time domain to the frequency domain. To extract the weak signal, we manually select the parameter values from the peak wave and apply Equation (A1). Any point on the wave crest can be used. In this scenario, we selected the point where $x_{0}$ = 0 and the associated *L* is 15.92 (at *f_approximate_ =* 10 Hz in the figure). The extracted signal and the corresponding spectrum are shown in Figure 6b–c. From the results, it is evident that the proposed approach has an excellent capacity for detecting feeble signals. 

**Figure 7:**
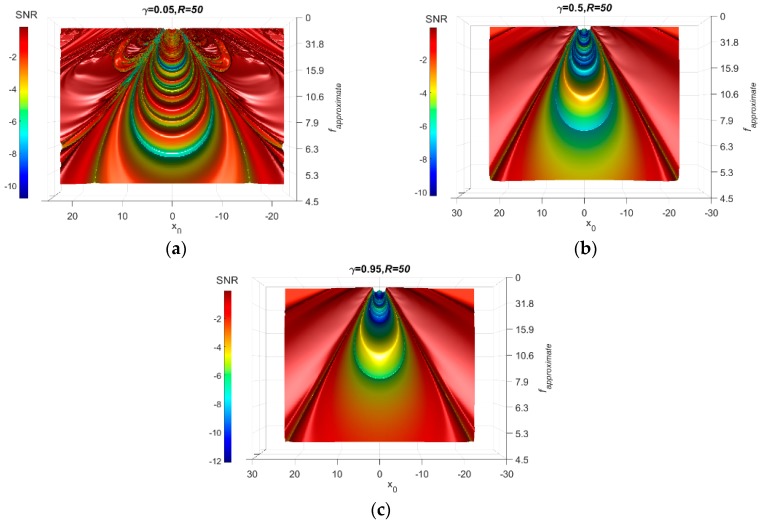
Influence of damping on the FI-UPRS method. (**a**) γ = 0.05 (**b**) γ = 0.5 (**c**) γ = 0.95.

**Figure 8:**
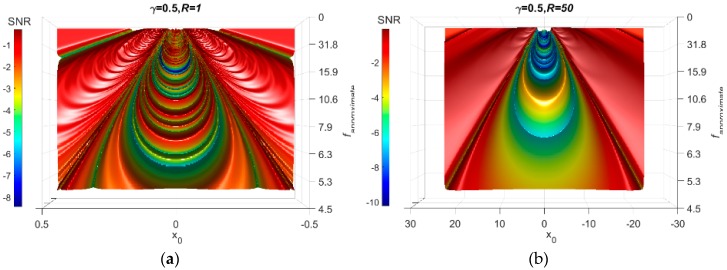

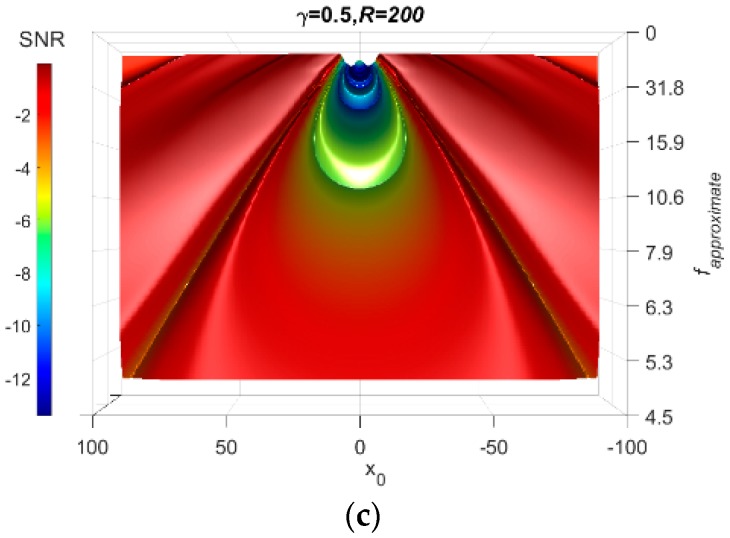
Influence of the scaling factor on the FI-UPRS method. (**a**) R = 1 (**b**) R = 50 (**c**) R = 200.

Returning back to the damping term, γ, it smooths the oscillation of the particle between the potential minima. Low damping coefficients will result in a stronger oscillation that develops spurious peaks in the SNR-Surface plot and, therefore, observing the weak signal in the SNR-surface plot becomes challenging. On the other hand, high damping coefficients severely damp the particle movement, which might affect the properties of the signal. Accordingly, a probable selection for the damping term is important. This can be performed by testing the SNR plots for different damping values. For the previous problem, the damping is set equal to 0.05, 0.5, and 0.95. The results are shown in Figure 7. The scaling factor *R* has a similar effect. Figure 8 presents the SNR plots for γ = 0.5 when *R =* 1, 50, and 200. For bridge structures, the recommended value for γ is between 0.08–0.2. For other infrastructures, further investigation is needed to determine the recommended ranges for γ. 

## 3. Extract Bridge Frequency from a Fast Passing Vehicle Signal Using FI-UPSR

**Figure 9:**
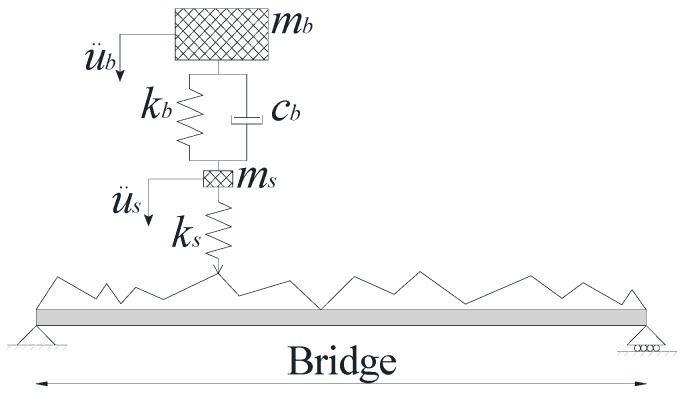
Theoretical quarter car model.

**Figure 10:**
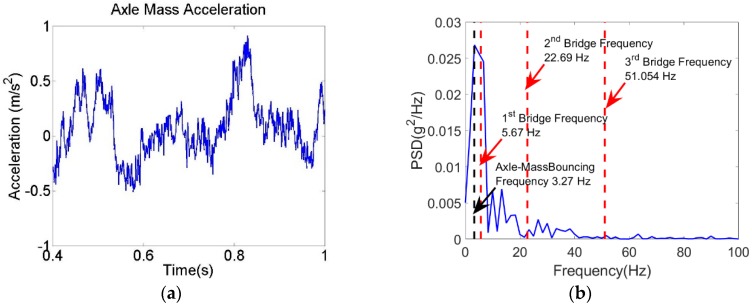
(**a**) Axle mass acceleration and (**b**) spectrum of the axle mass acceleration.

To numerically examine the proposed method, a Vehicle Bridge Interaction model [53] has been adopted to simulate the vehicle crossing over the bridge. The VBI model has been built using the fully-integrated Euler-Bernoulli Beam element, which has two degrees of freedom per node (vertical displacement and out of plane rotation). Axial displacement is omitted to avoid membrane locking. The Implicit Newmark-Beta integration scheme is utilized to solve the VBI equation of motion. A numerical damping is added to the scheme to suppress instabilities of the higher mode responses. The time step is set equal to 0.0005 s to capture the changes in the vehicle acceleration when it traverses the bridge. The vehicle and the bridge properties are listed in Table 2 and Table 3, respectively. Figure 9 illustrates the adopted VBI model. The road roughness has been randomly generated using ISO-8608 [54] specification (Roughness class “A”). The vehicle speed is 25 m/s. The vehicle passes over a 10 m approach distance before crossing the bridge. The recorded acceleration has been contaminated by a 90 dB Gaussian Noise. This value is chosen to match the records of the sensor used in the field experiment [55]. The acceleration of the vehicle’s axle mass and its PSD are illustrated in Figure 10. As expected, the vehicle response overwhelms the spectrum. 

**Figure 11:**
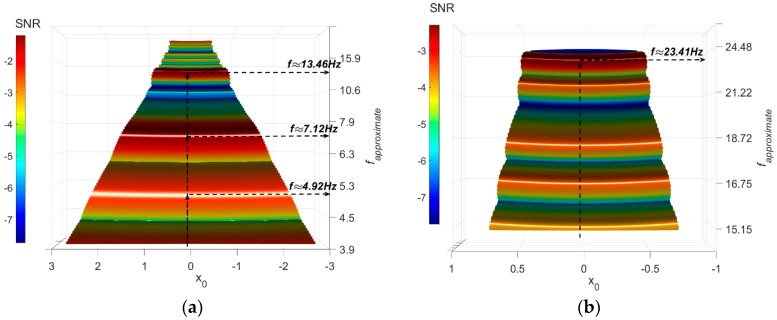
SNR-surface plots for the vehicle acceleration (**a**) [4 Hz–25 Hz]. (**b**) [15 Hz–25 Hz].

The axle acceleration has been processed using the FI-UPSR technique. Herein, *V_d_ =* 100, *R =* 50, and γ = 0.1 while the values of *x*_0_ and *L* have been selected to provide a scanning window bounded by [4 Hz → 25 Hz]. The SNR surface plot is illustrated in Figure 11.

**Figure 12:**
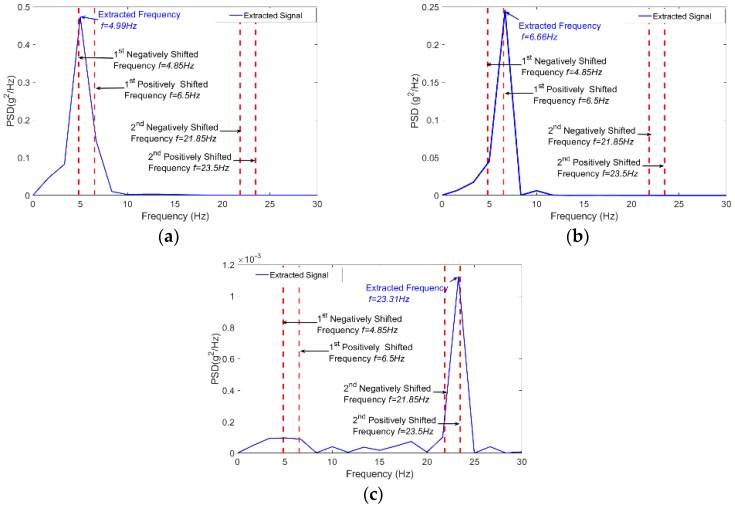
Extracted Signals PSD (**a**) first peak at *f* = 4.99 Hz, (**b**) second peak at *f* = 7.12 Hz, (**c**) third peak at *f* = 23.41 Hz.

The first point of note is that the road roughness and noise effects have been substantially removed and few peaks were kept in the SNR plot. In Figure 11a, the values of *f_approximate_* corresponding to the highest SNR are shown. The exact signals frequencies are identified by extracting the weak signals that correspond to the SNR peaks. Then plot the signal PSD depicted in Figure 12.

Two values have been determined for the first bridge frequency. Both values relate to the first bridge frequency, shifted by ± half the driving-frequency [17,18] (5.67 ± $\frac{25}{2\times 15}=4.84$ and 6.5). It is evident in the plot that the 6.5 Hz frequency has more power than the 4.84 Hz frequency. An ambiguous dominant peak is visible in the plot and is associated with a frequency of 13.32 Hz. This is apparently due to the influence of the road roughness. Thus, the approach could not totally attenuate the road roughness effect at higher frequencies. 

**Figure 13:**
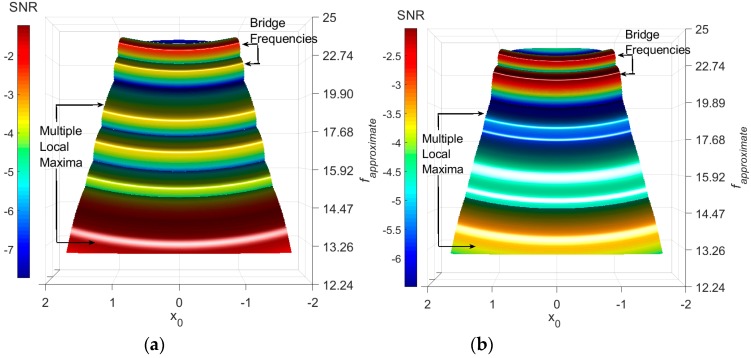
SNR-Surface plot for road profile Class “A” with (**a**) 2.5-cm increment (**b**) 25-cm increment.

**Figure 14:**
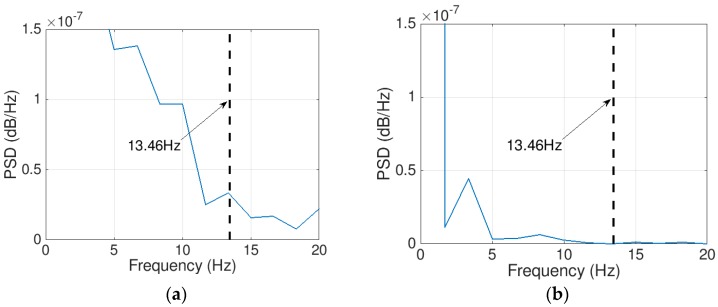
PSD plots for the contact point displacement history of the quarter car: (**a**) roughness is defined using a 2.5-cm increment (**b**) roughness is defined using a 25-cm increment.

The results suggest that the FI-UPSR is capable of detecting the lower bridge frequencies from a fast crossing vehicle measurement. However, the existence of an obscure frequency (13.46 Hz) requires a plausible interpretation to firmly root the approach before expanding it to the field scale. The authors presume that this is due to the nature of the representation for the contact between the vehicle and the road. The simplified Quarter-Car model touches the road at a single point. In tandem with that, the used road roughness is defined each 2.5 cm along the vehicle path. Thus, the vehicle will observe a stronger vibration due to the instantaneous change in the profile. This vibration produces multiple local maxima in the SNR surface plot especially at the higher frequencies, as presented in Figure 13a. In the field, on the other hand, the contact area between the vehicle tires and road roughness will attenuate the roughness effect on the vehicle signal. To simulate the condition in the field, the road roughness has been regenerated using a 25-cm increment. Figure 14 presents the PSD plots for the displacement history of the quarter car contact point and for the two scenarios (the 2.5-cm increment in Figure 14a and the 25-cm increment in Figure 14b). It is evident that increasing the length of the contact attenuates the effect of the road roughness on the vehicle vibration. The FI-UPSR surface plot has been regenerated utilizing the new vehicle acceleration. The results are presented in Figure 13b. The figure shows that, for milder profiles, local maxima have less power. This point requires further investigation to clearly depict the effect of the contact area between the vehicle and the road on the Drive-by Bridge Inspection approaches.

Regardless of this point, the influence of road roughness in exciting the vehicle and the dominance of the vehicle frequencies was overcome in this example for the first and the second bridge frequency. 

## 4. FI-UPSR Fidelity for Full Scale Field Test Data

**Figure 15:**
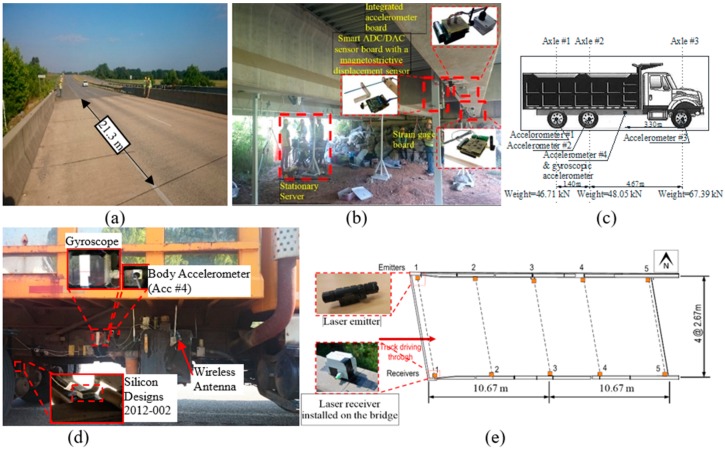
Field instrumentation, (**a**) bridge overview, (**b**) bridge instrumentation, (**c**) truck configuration and axle weights, (**d**) truck instrumentation, and (**e**) laser emitter layout.

The field test was carried out on a skewed pre-stressed bridge consisting of three simply supported spans. The bridge is located on HWY 113 in Bartow County, Atlanta, Georgia between Covered Bridge Rd. and Dry Creek Rd. Each span is 21.3 m from the centers of the two supports. The bridge deck is a reinforced concrete slab integral with five pre-stressed concrete girders. The roadway facility consists of two-lane one-way traffic and one hard shoulder. In addition to vehicle instrumentation, sensors were installed on the first span of the bridge (from the traffic direction) as shown in Figure 15a,b. The inspection vehicle used was instrumented with three Silicon Design 2012-002 accelerometers on its axles. A special accelerometer, which was developed by Dong, Zhu [56], was used to measure the body mass acceleration. This sensor was mounted near the vehicle’s center of gravity, which was illustrated in Figure 15c,d. At the same location, a gyroscope (ADXRS624) was installed to monitor the vehicle pitching motion. All the recorded data during the test were sent wirelessly to a DAQ station, which lies under the bridge. Furthermore, the bridge barriers were equipped with five laser emitters, which were utilized to determine the exact time when the vehicle entered and exited the bridge.

**Figure 16:**
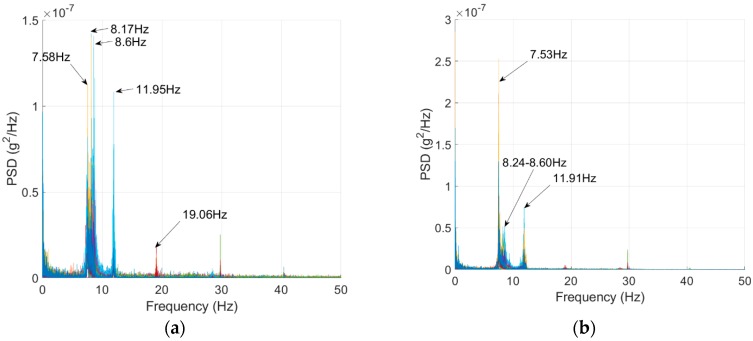
Acceleration PSDs for the Bridge Vibration Tests. (**a**) Test 1 and (**b**) Test 2.

The bridge natural frequencies were first identified by using a vibration test. This used the ELECTRO-SEIS Long Stroke Exciter vibration shaker while the bridge acceleration was recorded using 15 accelerometers installed on the girders (three sensors spaced equally on each of the bridge’s five girders). The sampling frequency was set to 100 Hz while the frequency resolution was 6.25 × 10^−3^ Hz. The traffic was blocked during the test to halt the traffic’s mass interference with the calculated frequency, which was noted by Yang, Cheng [57]. The test was repeated twice, and the spectra are illustrated in Figure 16. The bridge frequencies are listed in Table 4.

**Figure 17:**
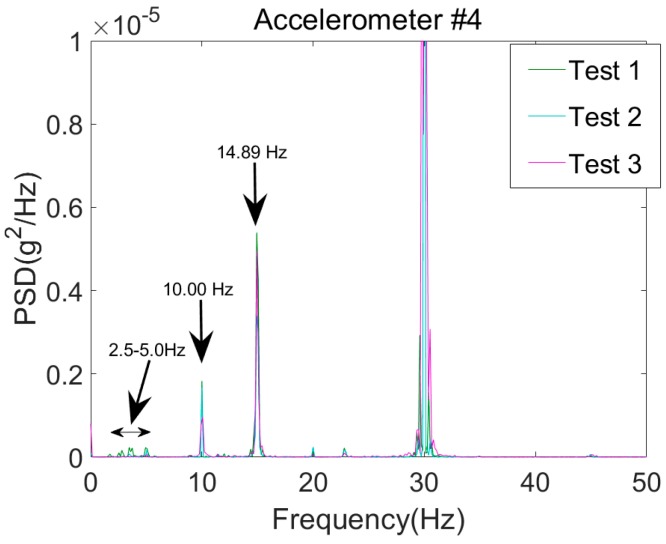
Acceleration spectra for the vehicle sensor.

**Figure 18:**
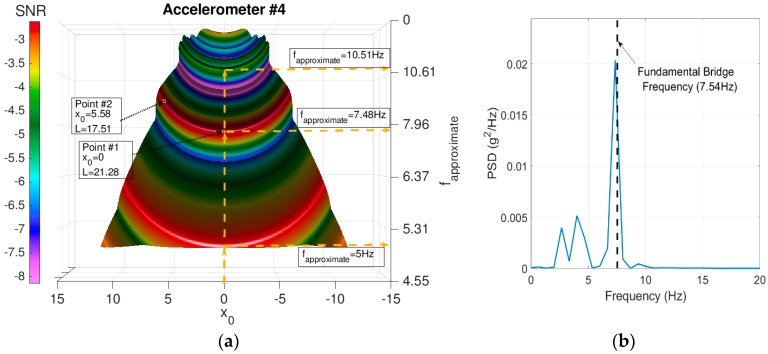
Test 3 results (**a**) FI-UPSR surface plot. (**b**) PSD for the extracted signal at *f_approximate_* = 7.48 Hz.

The inspection truck crossed the bridge three times at the speeds listed in Table 5. The truck could not reach the road’s speed limit (90 km/h or 60 mph) since the available approach distance was limited and the truck did not have enough time to accelerate. However, the maximum speed (i.e., for Test 3) is approximately 30% higher than the speed where the bridge frequencies are detectable (i.e., 40 km/h), which was previously introduced in the literature. Each test has two stages. (i) Stationary stage with engine running and (ii) moving stage where the vehicle accelerates to cross the bridge. The recorded accelerations when the truck was stationary and excited only by the vibration of the engine have been used to determine the frequencies of the vehicle mechanical system. The PSD for body mass acceleration (i.e., Accelerometer #4) is presented in Figure 17. The vehicle frequencies are identified using the acceleration spectrum. It is important to identify the vehicle frequencies to distinguish the bridge frequencies in the FI-UPSR plots. The frequency band is set to [5 Hz → 15 Hz]. The proposed FI-UPSR technique was utilized to process the data of the three tests for this frequency band. The SNR plots for Test 3 are presented in Figure 18a.

**Figure 19:**
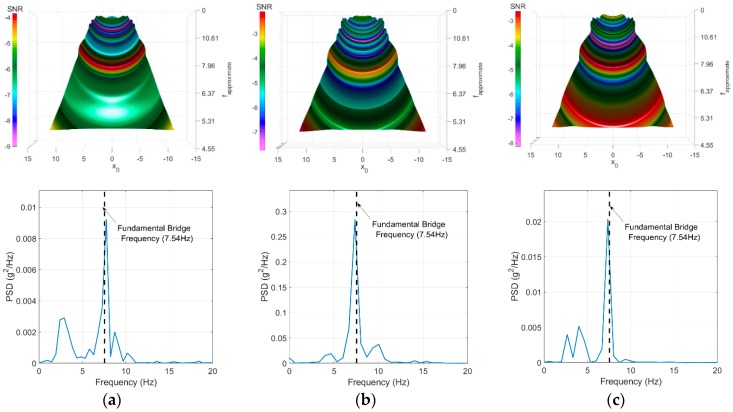
FI-UPSR SNR surface plots and signals PSD for the three tests. (**a**) Test 1, (**b**) Test 2, and (**c**) Test 3.

Three dominant peaks are observed in the surface plot. The peaks at 5 Hz and 10.5 Hz indicate the frequencies of the vehicle. The third peak refers to the first fundamental frequency for the bridge (the peak at *f_approximate_* = 7.48 Hz). To extract the bridge signal, we manually selected the potential parameter values from the crest of the peak-wave and then we applied Equation (A1). Any point on the crest of the peak wave can be utilized. For instance, Point #1 where *x*_0_ = 0 and *L* = 21.28 or Point #2 where *x*_0_ = 5.58 and *L* = 17.51. In this example, we utilized the coordinates of Point #1. The PSD for the extracted signal is presented in Figure 18b. The figure reveals a successful identification for the first bridge frequency. The same surface plot attribute is observed for Test 1 and Test 2. The results for Test 1 and Test 2 are presented in Figure 19a,b. 

The results presented in the figure show a successful identification for the first bridge frequency for all three tests. The second frequency has not been observed in any of the surface plots. This may be because the second mode of vibration (i.e., a transverse mode of vibration) interferes less with the body mass vibration. The third frequency has not been successfully extracted from the vehicle responses. The authors presume that the frequency content of the vehicle (10.00 Hz) and the bridge frequency (11.93 Hz) have been fused to provide an average peak between them. This is shown in Figure 19 where the peak takes place between 10.0 Hz to 12.00 Hz.

While the results of the field test demonstrated a successful identification for the first bridge frequency, the SNR-surface plot exhibit multiple local maxima due to the vehicle frequencies that make the bridge frequency identification more challenging. The authors suggest that adopting a truck trailer model where the sensors are installed in the trailer and the trailer suspension properties are chosen carefully to provide low-frequency content may minimize the interference of the vehicle frequencies and positively affect the approach.

Another point of note is the impact of the environmental condition on the accuracy of assessing the bridge frequencies utilizing the drive-by approach. It is often the influence of temperature on the stiffness of the bridge, which results in changes in natural frequency. This point can be addressed by finding or estimating the temperature of the bridge and, hence, applying a correction where the temperature equipment could be mounted on the vehicle to perform the correction instantaneously. Another option is to mitigate the influence of temperature by choosing the best times of day for screening to minimize temperature differences from one test to the other. However, these issues present when utilizing short-time measurements of frequency to monitor the bridge condition.

**Figure 20:**
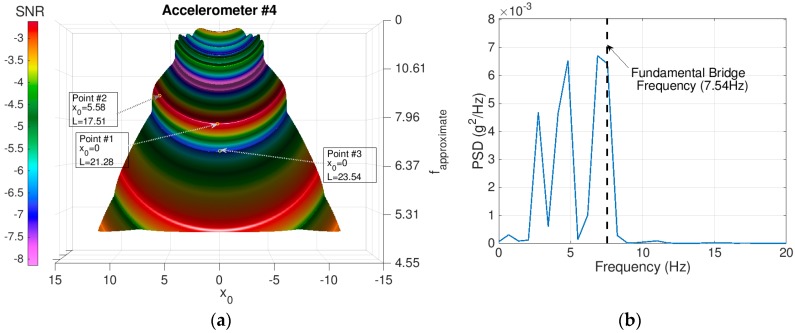
The example illustrates the effect of poor selection of potential parameters on PSD for the extracted signal. (**a**) FI-UPSR surface plot for Test #3 and (**b**) PSD plot for extracted signal using the potential parameters for Point #3.

While the approach holds significant merits and its application can be expanded to other SHM problems, the utilization of a visual peak picking process subjects the results to some uncertainty. The process requires a priori experience about the SNR surface plot shapes to properly identify the peaks. Figure 20b demonstrates the PSD for the extracted signal for Test #3 where the point is taken from the wave trough rather than the wave crest (Point #3 at Figure 20a). As presented in the figure, spurious peaks start to dominate the PSD plot and, therefore, it becomes unviable for identifying the bridge frequency. Further work is needed to implement a suitable peak-picking algorithm to automatically identify the peaks within a certain frequency band.

## 5. Conclusions

Recently, the drive-by bridge inspection has become a burgeoning field of research in the bridge health-monitoring domain. However, it faces a major obstacle in the need for low speeds of the inspection vehicle. This paper introduces Frequency Independent Underdamped Pinning Stochastic Resonance (FI-UPSR) as a new technique to extract the dynamic characteristics of the bridge from the responses of a passing vehicle operating with a high speed. Stochastic Resonance is a process for the detection of a feeble property in a very rough domain through the addition of noise. This paper invests the UPSR model developed by Zhang, He [50] as a suitable model for Civil structures since it works with mono-stable/underdamped systems, which are features of most of these structures. The UPSR model requires a priori knowledge of the targeted frequency to extract the waveform associated with it. Therefore, the authors have introduced a graphical approach that can be utilized to identify feeble signals in a response disrupted by severe noise. The new approach is based on the plotting of 2D surface plots of SNR values for a specified range of UPSR parameters. By visualizing the plots, the values of the parameters that provide the maximum SNR can be observed and, hence, the weak signals can be extracted. The article has explored numerically the use of FI-UPSR for the extraction of feeble bridge vibrations from a fast passing vehicle and the results have revealed a successful identification of the first and second bridge frequencies. A numerical VBI MATLAB model has been used to simulate the crossing of the vehicle over the bridge at 25 m/s (90 km/h). 

To investigate the fidelity of the approach in practice, a full-scale field test has been utilized. The experiment employed an inspection truck equipped with an accelerometer on its center of gravity to extract the fundamental bridge frequencies. First, the data from 15 accelerometers installed on the bridge were used to identify its fundamental frequencies. Afterwards, the inspection truck crossed the bridge at three different speeds. The data of the truck accelerometers have been processed using the FI-UPSR method to identify the bridge frequencies. The results show a successful identification of the first natural frequency for the bridge of all test speeds. The second mode of vibration for the bridge is orthogonal to the traffic direction and, therefore, it was not evident in the vehicle body mass acceleration history. 

The proposed FI-UPSR method shows good potential to amplify a weak property in a noisy disrupted signal, which could have significant applications in Civil SHM. In conclusion, the paper presents an alternative methodology to process the data of the vehicle sensors by using rather than suppressing unwanted noise in the recorded signal. Further investigation is required on the impact of environmental effects on the proposed approach.

## Figures and Tables

**Table 1:** Recommended initial values for *V_d_*, *R*, and *γ.*

*V_d_*	R	γ
≥100	25–75	0.1–0.7
	For dt = 0.01–0.0005	

**Table 2:** Properties of the quarter car model.

Property	Unit	Symbol	Value
Body Mass	kg	*m_b_*	16,600
Axle Mass	kg	*m_s_*	700
Body Stiffness	N/m	*k_b_*	2 × 10^4^
Body Damping	N.s/m	*c_b_*	10 × 10^3^
Suspension Stiffness	N/m	*k_s_*	2.75 × 10^5^
Body Bounce Frequency	Hz	*f_bounce_*	0.169
Axle Hop Frequency	Hz	*f_axle_*	3.27

**Table 3:** Properties of the bridge.

Property	Unit	Value
Length	m	15
Mass Per Unit Length	kg/m	28,125
Elastic Modulus	MPa	35,000
Second Moment of Area	m^4^	0.5273
1st Frequency	Hz	5.67
2nd Frequency	Hz	22.69
3rd Frequency	Hz	51.05

**Table 4:** Average bridge frequency (Hz).

1st	2nd	3rd	4th
7.56	8.4	11.93	19.04

**Table 5:** Vehicle speeds km/h.

Test 1	Test 2	Test 3
37	51	53

## Appendix A

The appendix presents the discretized version of Equation (1) developed by Zhang, He [50].

(A1) $$\begin{array}{c} \;V^{\prime }\left(x\right)=\frac{dV\left(x\right)}{dx}=V_{d}\left(\frac{2\left(x+x_{0}\right)}{L^{2}}\exp\left(-\frac{{\left(x+x_{0}\right)}^{2}}{L^{2}}\right)+\frac{2\left(x-x_{0}\right)}{L^{2}}\exp\left(-\frac{\left(x-x_{0}\right)}{L^{2}}\right)\right)\\ x\left(0\right)=0;\;\frac{dx}{dt}\left(0\right)=0\\ \mathrm{For}\;i=2:\;\mathrm{end}\\ k_{x1}=\frac{dx}{dt}\left(i-1\right)\\ k_{y1}=-V^{\prime }\left(x\left(i-1\right)\right)-\gamma \frac{dx}{dt}\left(i-1\right)+\left\{s\left(i-1\right)+n\left(i-1\right)\right\}\;\\ k_{x2}=\frac{dx}{dt}\left(i-1\right)+\frac{h}{2}k_{y1}\\ k_{y2}=-V^{\prime }\left(x\left(i-1\right)+\frac{h}{2}k_{x1}\right)-\gamma \left(\frac{dx}{dt}\left(i-1\right)+\frac{h}{2}k_{y1}\right)+\left\{s\left(i-1\right)+n\left(i-1\right)\right\}\;\\ k_{x3}=\frac{dx}{dt}\left(i-1\right)+\frac{h}{2}k_{y2}\\ k_{y3}=-V^{\prime }\left(x\left(i-1\right)+\frac{h}{2}k_{x2}\right)-\gamma \left(\frac{dx}{dt}\left(i-1\right)+\frac{h}{2}k_{y2}\right)+\left\{s\left(i\right)+n\left(i\right)\right\}\;\\ k_{x4}=\frac{dx}{dt}\left(i-1\right)+hk_{y3}\\ k_{y4}=-V^{\prime }\left(x\left(i-1\right)+hk_{x3}\right)-\gamma \left(\frac{dx}{dt}\left(i-1\right)+hk_{y3}\right)+\left\{s\left(i\right)+n\left(i\right)\right\}\;\\ x\left(i\right)=x\left(i-1\right)+\frac{h}{6}\left(k_{x1}+2k_{x2}+2k_{x3}+k_{x4}\right)\\ \frac{dx}{dt}\left(i\right)=\frac{dx}{dt}\left(i-1\right)+\frac{h}{6}\left(k_{y1}+2k_{y2}+2k_{y3}+k_{y4}\right) \end{array}$$

where *h* is the calculation step and equals *R*dt* (*dt* is the time step and *R* is a rescaling factor). The parameter, *x*(*t*), is the targeted weak property or, in this case, the weak signal.

## Appendix B

This section presents the derivation of the approximate relationship between the approximate frequency *f_approximate_* and the potential parameters of the UPSR model. The original SR equation is shown below.

(A2) $$\frac{d^{2}x}{dt^{2}}=-V^{\prime }\left(x\right)-\gamma \frac{dx}{dt}+\left\{s\left(t\right)+n\left(t\right)\right\}$$

(A3) $$\frac{d^{2}x}{dt^{2}}+\gamma \frac{dx}{dt}+V^{\prime }\left(x\right)=\left\{s\left(t\right)+n\left(t\right)\right\}$$

Knowing that:

(A4) $$V\left(x\right)=V_{0}-V_{d}\left(\exp\left(-\frac{{\left(x+x_{0}\right)}^{2}}{L^{2}}\right)+\exp\left(-\frac{{\left(x-x_{0}\right)}^{2}}{L^{2}}\right)\right)$$

Then,

(A5) $$V^{\prime }\left(x\right)=\frac{dV\left(x\right)}{dx}=V_{d}\left(\frac{2\left(x+x_{0}\right)}{L^{2}}\exp\left(-\frac{{\left(x+x_{0}\right)}^{2}}{L^{2}}\right)+\frac{2\left(x-x_{0}\right)}{L^{2}}\exp\left(-\frac{\left(x-x_{0}\right)}{L^{2}}\right)\right)$$

The SNR plots in Section 2 and Section 3 illustrate the essential features of the UPSR model. First, for the mono-stable zone, the SNR plot is symmetric around *x*_0_ *=* 0. In addition, the SNR peaks are more evident around *x*_0_ *=* 0. Furthermore, Equation (A5) shows that, for specific values of *x*_0_, increasing *V_d_* requires an increase in *L* to extract the same signal *x*(*t*) by balancing the $V_{d}/L^{2}$ ratio. Accordingly, use *x*_0_ *=* 0 and a considerably high value for *L* to simplify the formula. Thus, Equation (A5) yields the following.

(A6) $$V^{\prime }\left(x\right)\approx \frac{4V_{d}}{L^{2}}x\exp\left(-\frac{x^{2}}{L^{2}}\right)$$

$$\mathrm{For}\;\mathrm{large}\;L,\;\exp\left(-\frac{x^{2}}{L^{2}}\right)\approx 1$$

Hence,

(A7) $$V^{\prime }\left(x\right)\approx \frac{4V_{d}}{L^{2}}x$$

Substituting in Equation (A3) then gives the equation below.

(A8) $$\frac{d^{2}x}{dt^{2}}+\gamma \frac{dx}{dt}+\frac{4V_{d}}{L^{2}}x=\left\{s\left(t\right)+n\left(t\right)\right\}$$

The equation yields to the form of a forced damped vibration for a single degree of freedom system where the system will resonate when the forcing frequency ({s(t)+n(t)}) matches the system frequency. The system frequency for the left hand side, neglecting the damping term for simplicity, equals the following.

(A9) $$\omega =2\pi f_{approximate}=\sqrt{\frac{4V_{d}}{L^{2}}}$$

Thus:

(A10) $$f_{approximate}=\sqrt{\frac{V_{d}}{\pi^{2}L^{2}}}$$

It is worth mentioning that the time step for the discretized equation, as depicted in Equation (A1), is rescaled by a factor *R.* To consider this, the frequency needs to be re-scaled with 1/*R*, which is shown below.

(A11) $$\frac{f_{approximate}}{R}=\sqrt{\frac{V_{d}}{\pi^{2}L^{2}}}$$
